# Scalable Engineering of Superhydrophobic Copper Surfaces with Enhanced Corrosion Resistance by Combined Nanostructuring and Chemical Vapor Deposition

**DOI:** 10.3390/ma18173981

**Published:** 2025-08-25

**Authors:** N. Rahul, Beomguk Park, Sanjaya Kumar Pradhan, Ho-Eon Sung, Inn-Hyup Jeong, Yong-Sup Yun, Min-Suk Oh

**Affiliations:** 1 Division of Advanced Materials Engineering, Department of Energy Storage and Conversion Engineering, Graduate School, Jeonbuk National University, Jeonju 54896, Republic of Korea; 2 R&D Department, MOLIT Co., Ltd., Hanam 12930, Republic of Korea; beomguk@gmail.com; 3 Korea Institute of Corrosion Science & Technology, National Korea Maritime and Ocean University, Busan 49112, Republic of Korea

**Keywords:** superhydrophobic, corrosion, chemical vapor deposition, thin film, interfacial phenomena

## Abstract

The vulnerability of copper to corrosion in humid and saline environments remains a critical challenge for its long-term use. In this work, we present a streamlined and scalable approach for fabricating superhydrophobic, corrosion-resistant copper surfaces by integrating a simple wet chemical oxidation process with atmospheric pressure chemical vapor deposition (APCVD) of a perfluorinated silane. The hierarchical CuO nanostructures formed via alkaline oxidation serve as a robust layer, while subsequent silane functionalization imparts low surface energy, resulting in surfaces with water contact angles exceeding 170° and minimal contact angle hysteresis. Comprehensive surface characterization by SEM and roughness analysis confirmed the preservation of hierarchical morphology after coating. Wettability studies reveal a transition from hydrophilic to superhydrophobic behavior, with the Cassie–Baxter regime achieved on nanostructured and silane-functionalized samples, leading to enhanced droplet mobility and self-cleaning effect. Salt spray tests demonstrate that the superhydrophobic surfaces exhibit a corrosion rate reduction of 85.7% (from 2.51 mm/year for bare copper to 0.36 mm/year for the treated surface), indicating a seven-fold improvement in corrosion resistance compared to bare copper. This methodology offers a practical, reproducible route to multifunctional copper surfaces, advancing their potential for use in anti-fouling, self-cleaning, and long-term protective applications.

## 1. Introduction

Copper’s widespread use in electronics, energy, and marine applications is often limited by its vulnerability to corrosion, particularly in chloride-rich and humid environments [1,2,3]. This persistent challenge has driven the search for advanced surface engineering strategies that can impart both corrosion resistance and additional functionalities, such as self-cleaning and anti-fouling behavior [4]. Superhydrophobic surfaces, defined by water contact angles above 150° and low contact angle hysteresis, have attracted significant attention as a potential solution [5,6,7], as they minimize the interaction between water and the underlying metal, thereby reducing the risk of corrosion and fouling [8]. The scientific foundation for these surfaces lies in classical wetting theories [9], where the combination of hierarchical surface roughness and low-surface-energy chemical modification is essential for achieving extreme water repellency and robust droplet mobility [10,11].

Recent advances in materials engineering have shown that designing micro- and nanoscale features on copper surfaces, followed by chemical functionalization with hydrophobic agents, can impart exceptional water repellency and significantly enhance corrosion resistance. Techniques such as wet chemical oxidation and etching have been widely employed to fabricate copper oxide (CuO or Cu_2_O) nanostructures, which act as an effective layer for further modification with low-surface-energy compounds such as fluorinated silanes or thiols, resulting in water contact angles often exceeding 160° and strong reductions in corrosion rates [12,13,14]. Electrodeposition strategies have also been reported for creating robust, multiscale roughness; when combined with hydrophobic coatings, these approaches further boost corrosion-resistant properties and mechanical durability [15]. Chemical vapor deposition (CVD) methods have enabled the conformal deposition of organosilane or fluoropolymer layers onto structured copper, producing superhydrophobic coatings that retain their functionality under prolonged exposure to aggressive environments [16,17,18].

In recent years, the development of green, wear-resistant, and antibacterial superhydrophobic coatings on copper and its alloys has expanded the field, demonstrating multifunctional surfaces that deliver not only enhanced corrosion resistance but also durability and potential for pathogen neutralization [19]. In-situ methods like anodization, as well as facile reduction-based fabrication of copper nanoparticles, have led to cost-effective and scalable solutions for achieving superhydrophobicity on a variety of substrates, often with self-cleaning, anti-icing, and oil/water separation capabilities [20,21]. These strategies all rely on the principle that hierarchical micro- and nanoscale surface structuring traps air beneath liquid drops, minimizing the solid–liquid contact area and effectively impeding the ingress of corrosive species [22]. While the literature documents several hundred approaches for engineering superhydrophobic copper coatings, most reported methods remain complex, require specialized equipment, involve toxic agents, or are not easily scalable for industrial use. In particular, few studies demonstrate a process that simultaneously preserves hierarchical CuO nanostructures during coating, achieves Cassie–Baxter wetting with water contact angles exceeding 170° and minimal hysteresis, and provides long-term corrosion resistance under aggressive saline conditions with corrosion rates below 0.4 mm/year. Achieving a balance between mechanical robustness, long-term stability, and multifunctionality is still a significant challenge in the field. Motivated by these limitations, the present work introduces a reproducible approach for fabricating superhydrophobic, corrosion-resistant copper surfaces. Our methodology integrates a simple wet chemical oxidation process to generate robust CuO nanostructures with a chemical vapor deposition-based silane functionalization, which forms a uniform, low-surface-energy coating. Unlike many previously reported methods, our process preserves the hierarchical morphology while ensuring complete and conformal silane coverage, resulting in surfaces with minimal contact angle hysteresis and significantly improved resistance to salt-induced corrosion. The resulting superhydrophobic copper surfaces not only demonstrate exceptional water repellency and droplet mobility but also show enhanced durability against corrosion in harsh saline environments. This practical, scalable fabrication route offers a significant advance over previous approaches, making it feasible to produce multifunctional copper surfaces for a wide range of technological applications, including anti-fouling, self-cleaning, and long-term protection in challenging operational settings. Our findings contribute to the broader understanding of structure–property relationships in engineered interfaces and provide a valuable framework for the design of next-generation, high-performance copper-based materials.

## 2. Materials and Methods

**Figure 1:**
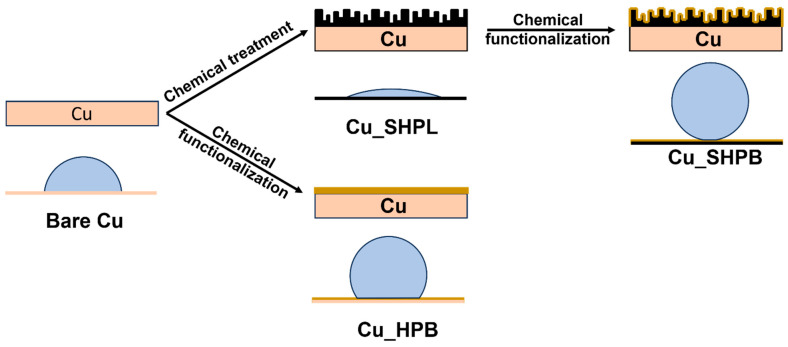
Schematic illustration of the wettability of the copper substrate and surface modification to achieve non-wetting surfaces.

The schematic in Figure 1 illustrates the wetting states of the bare copper substrate and the modified superhydrophobic surface, showing the transition from complete wetting to the Cassie–Baxter regime. This visual guide highlights the role of hierarchical nanostructuring and chemical functionalization in achieving non-wetting behavior. Copper substrates were used for all experiments. The substrates were initially cleaned by sonication in a bath sonicator. To remove surface contaminants, the copper samples were sonicated in acetone for 10 min, followed sequentially by cleaning with ethanol and deionized (DI) water. To eliminate the native oxide layer, the cleaned copper surfaces were immersed in a 2 M HCl solution for 10 min, immediately rinsed with DI water, and gently wiped with tissue to minimize re-oxidation. To fabricate copper oxide nanostructures, an alkaline solution comprising NaClO_2_, NaOH, Na_3_PO_4_·12H_2_O, (Chemicals purchased from Sigma-Aldrich, Taufkirchen, Germany) and DI water (in weight ratios of 3.75:5:10:100, respectively) was prepared and maintained at 97 ± 3 °C.

**Figure 2:**
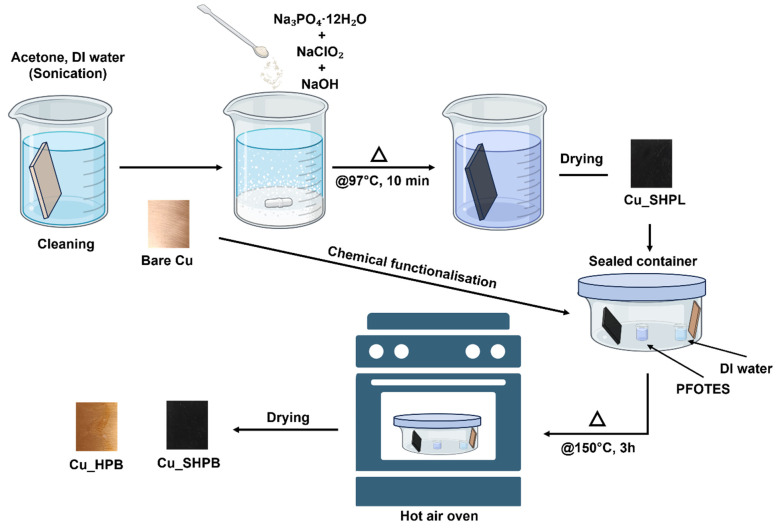
Schematic representation of the experimental methodology for fabricating various surfaces.

The copper samples were immersed in this solution for 10 min, resulting in the formation of a thin Cu_2_O layer (~500 nm), which subsequently oxidized further to yield copper oxide nanostructures approximately 1 µm thick. For chemical functionalization, atmospheric pressure chemical vapor deposition (APCVD) was employed using 1H,1H,2H,2H-perfluorooctyltriethoxysilane (PFOTES) purchased from sigma-aldrich (Bellefonte, PA, USA) and ethanol in a 1:9 volume ratio. A 1 mL aliquot of the PFOTES-ethanol solution (10% *v*/*v*) was placed in a small glass beaker inside a crystallizing dish alongside the copper samples as shown in Figure 2. To facilitate surface hydrolysis, an additional beaker containing 5 mL DI water was also placed in the dish. The container was sealed with aluminum foil and placed in a hot air oven at 150 °C for 4 h, allowing the silane to evaporate and deposit onto the copper surface, forming a uniform monolayer coating.

### 2.1. Surface Characterization

Surface morphology of the samples was characterized using field-emission scanning electron microscopy (FE-SEM, SUPRA40VP, Zeiss, Oberkochen, Germany) operated in secondary electron (SE) mode, enabling detailed visualization of micro- and nanoscale surface features. Surface roughness measurements were performed with a high-speed, three-dimensional (3D) laser confocal microscope (SURFVIEW-STANDARD, GLTech, Hwaseong, Republic of Korea), providing a quantitative assessment of the topographical variations and roughness parameters across the different surfaces.

Contact angle measurements were performed using a Phoenix-Pico/Nano (SEO) goniometer (Surface Electro Optics Co., Ltd., Hwaseong, Republic of Korea) with distilled water as the probe liquid. To ensure reliable and representative data, each copper sample (20 mm × 20 mm) was analyzed at three distinct locations, and the reported values represent the average of these measurements. Both advancing and receding contact angles, as well as static contact angles, were determined to assess the wetting behavior and surface hysteresis. A 5 μL sessile water droplet was carefully dispensed onto the sample surface for each measurement. The droplet images were captured in real time using a high-resolution CCD camera (Surface Electro Optics Co., Ltd., Hwaseong, Republic of Korea), and contact angles were quantified using Surfaceware 9 software. To evaluate surface wettability and characterize the dynamic behavior of the contact line, which is important for assessing droplet mobility and pinning effects on engineered copper surfaces.

X-ray diffraction (XRD, Rigaku RINT-2000, Hokuto City, Japan) measurements were used to detect the phases present in the uncoated and coated copper samples using monochromatic Cu–Kα radiation (*λ* = 1.5406 Å) in the 2θ range of 10°–80°. The step size and scanning speed were 0.02° and 2°/min, respectively.

### 2.2. Electrochemical Measurements

Electrochemical corrosion behavior was evaluated using a potentiostat (GAMRY INTERFACE 1010E, GAMRY Instruments, Warminster, PA, USA) employing both potentiodynamic polarization and electrochemical impedance spectroscopy (EIS) techniques. All measurements were performed in a conventional three-electrode electrochemical cell at room temperature. A saturated calomel electrode (SCE) served as the reference electrode, a graphite rod was used as the counter electrode, and the bare or coated copper samples acted as the working electrode. The exposed area of each working electrode was fixed at 1 cm^2^, and all experiments were conducted in a 3.5 wt% NaCl aqueous solution.

Prior to electrochemical testing, the open circuit potential (OCP) of each sample was monitored for 2 h to ensure stabilization. EIS measurements were then carried out at the stabilized OCP, applying a sinusoidal perturbation of 10 mV root mean square (rms) amplitude over a frequency range from 10^5^ Hz to 10^−2^ Hz. Potentiodynamic polarization curves were subsequently recorded at a scan rate of 0.5 mV/s, with the applied potential swept from −0.3 V to +0.5 V relative to the open circuit potential (E_ocp_).

$$IE=\frac{\left(R_{t}-R_{to}\right)}{R_{t}}\times 100$$

where R_t_ (R_t_ = R_ct_ + R_c_) is the charge transfer resistance of the modified Cu, and R_to_ is the resistance of the bare Cu.

Corrosion potential (E_corr_) and corrosion current density (i_corr_) values were determined by Tafel extrapolation of the polarization curves. EIS data fitting and analysis were performed using Gamry Echem Analysis software (version 7.10.0). All measurements were repeated at least three times to ensure reproducibility, and representative data are reported.

$$CR=0.13\frac{i_{\mathrm{corr}\;}\left(\frac{\mu A}{{cm}^{2}}\right)(EW)}{d}$$

**Figure 3:**
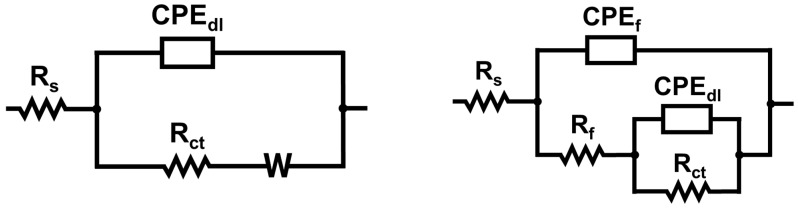
Equivalent circuit models for bare copper (on **left**) and coated copper surface (on **right**).

The EIS data were analyzed using an equivalent circuit model comprising solution resistance (R_s_), coating film resistance (R_f_), charge transfer resistance (R_ct_), constant phase elements for both the coating (CPE_f_) and double layer (CPE_dl_), and Warburg impedance (W), as illustrated in Figure 3.

The use of constant phase elements accounts for non-ideal capacitive behavior often observed at the electrode/electrolyte interface due to surface heterogeneity. The impedance of the CPE was calculated according to the following:

$$Z_{CPE}=Y_{0}^{-1}\cdot (j\cdot \omega {)}^{-n}$$

where Y_0_ is the magnitude of the CPE, ω is the angular frequency, and n is an exponent (0 < n < 1) describing the deviation from ideal capacitive behavior.

The corrosion inhibition efficiency (IE%) was calculated from the charge transfer resistance values using the following:

$$IE\%=\frac{R_{ct}^{\prime }-R_{ct}}{R_{ct}^{\prime }}\times 100\%$$

where R′_ct_ and R_ct_ are the charge transfer resistances with and without the coating, respectively. The corrosion rate (MPY) was calculated from i_corr_ using the standard relationship as follows:

$$\mathrm{Corrosion}\;\mathrm{Rate}\;(Mils\;per\;Year)=\frac{0.13\times i_{\mathrm{corr}\;}\times EW}{\rho }$$

where i_corr_ is in μA/cm^2^, EW is the equivalent weight of copper, and ρ is the density of copper.

### 2.3. Salt Spray Test

Corrosion resistance of the prepared copper surfaces was systematically evaluated using a salt spray tester (STP-90C-3, Suga, Hiroshima, Japan) in accordance with the ASTM B117 international standard [23]. Prior to testing, all specimens were thoroughly degreased with acetone, rinsed with distilled water, and air-dried to ensure a clean and uniform surface. The exposed surface area of each specimen was maintained at 4 × 6 cm.

A 5 wt% sodium chloride (NaCl) aqueous solution was used as the corrosive medium. The salt spray chamber was operated under controlled conditions: a spray rate of 1.5 ± 0.5 mL/h, atomizing air pressure of 0.9–1.1 bar, and a chamber temperature maintained at 35 ± 1 °C. The total exposure duration was set to 1440 h, with specimens periodically removed at designated intervals for analysis. At each interval, specimens were carefully rinsed with distilled water and dried. Visual inspections were conducted to qualitatively assess the progression of corrosion, including the formation of corrosion products and any changes in surface appearance. Quantitative evaluation was performed by measuring the initial mass (W_i_) prior to exposure and the final mass (W_f_) after each interval. The corrosion rate (CR) can be calculated based on the mass loss using the following relationship:

$$\mathrm{Corrosion}\;\mathrm{Rate}\;(mm/\;\mathrm{year})=\frac{87.6\times \left(W_{i}-W_{f}\right)}{\rho \times A\times t}$$

where W_i_ and W_f_ are the initial and final masses (mg), ρ is the density of copper (g/cm^3^), A is the exposed area (cm^2^), and t is the exposure time (hours). All tests were conducted in triplicate to ensure reproducibility, and representative results are reported.

## 3. Results and Discussion

### 3.1. Surface Characterization

**Figure 4:**
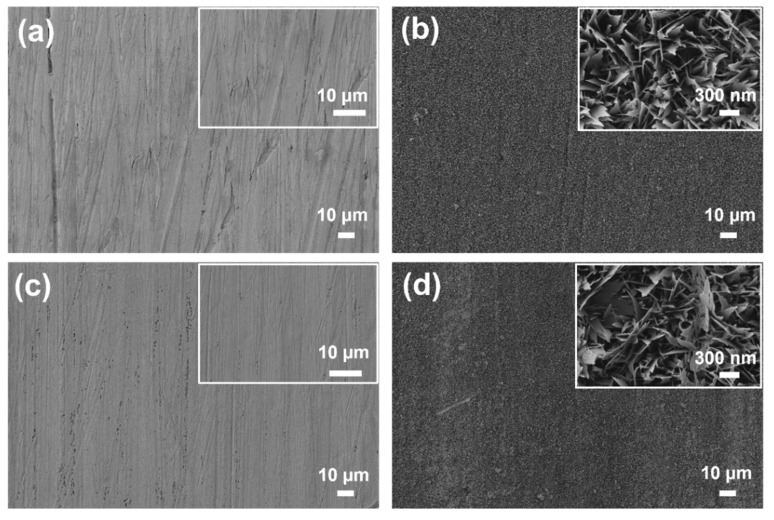
Scanning electron microscopy (SEM) images of copper surfaces at different modification stages: (**a**) Cu_Bare; (**b**) Cu_SHPL; (**c**) Cu_HPB; and (**d**) Cu_SHPB. Insets show high-magnification views of nanoscale features.

**Figure 5:**
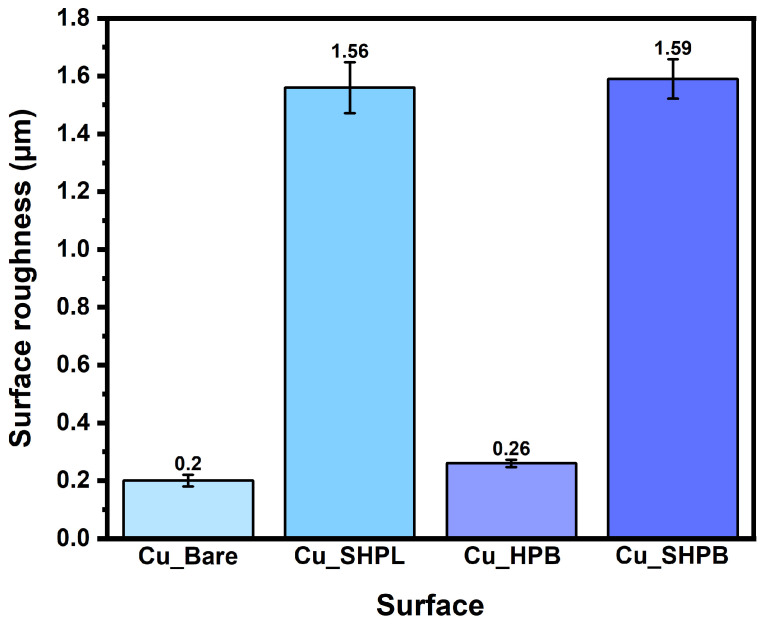
Quantitative comparison of surface roughness (Ra) for all samples.

The surface morphology of the copper substrates at various stages of modification is shown in Figure 4a–d. Bare copper exhibited a relatively smooth surface with visible polishing marks and an absence of distinct micro- or nanostructures, as confirmed by higher-magnification imaging. Following chemical oxidation in an alkaline solution, the Cu_SHPL surface transformed, displaying a dense layer of hierarchical nanostructures [15,16]. At higher magnification, these features appeared as vertically aligned, needle-like or grass-like CuO formations, which significantly increased the surface area and roughness as shown in Figure 5. Cross-sectional observation during sample preparation and literature-supported estimates for this oxidation method indicate that the nanostructures have an average height of approximately 1 µm atop an initial ~500 nm Cu_2_O base layer. This hierarchical oxide layer is stable, dense, and adherent, providing an effective scaffold for the subsequent conformal silane coating observed in the Cu_SHPB samples [15,16,22].

Subsequent chemical functionalization with PFOTES resulted in Cu_HPB that appeared uniformly smooth and featureless, indicating the presence of a thin, conformal silane layer that did not substantially alter the underlying topography. When the nanostructured copper was further coated with PFOTES (Cu_SHPB), the hierarchical morphology was retained, with the silane layer conformally covering the nanostructures [16]. High-magnification images confirmed that the nanostructured features remained intact, while the surface chemistry was modified by the low-surface-energy silane.

**Figure 6:**
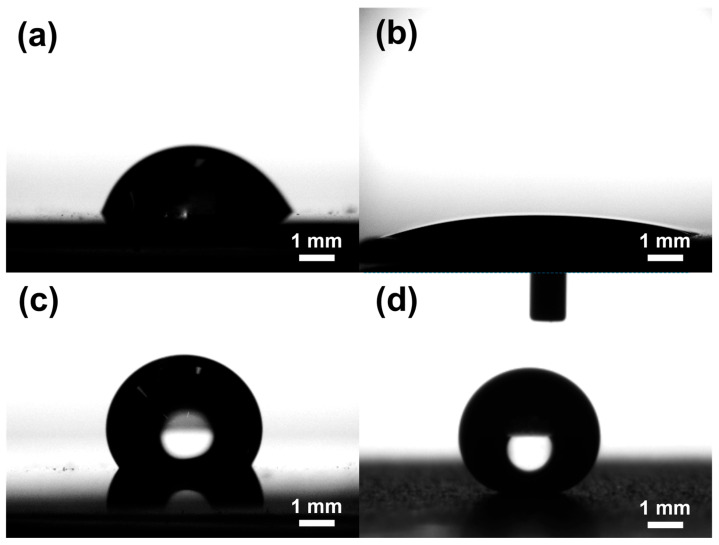
Static water contact angle images for (**a**) Cu_Bare, (**b**) Cu_SHPL, (**c**) Cu_HPB, and (**d**) Cu_SHPB.

**Figure 7:**
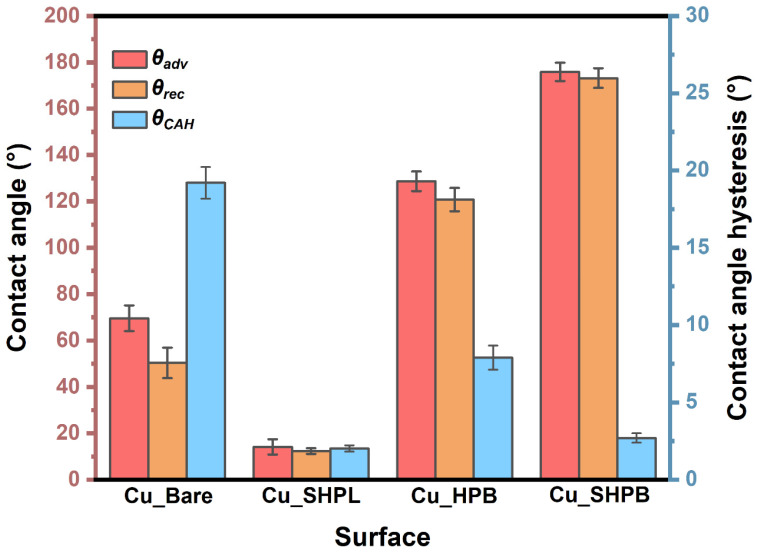
Contact angle and contact angle hysteresis measurements for all modified copper samples.

The bare copper surface exhibited moderate contact angles and high hysteresis, consistent with Young’s regime modified by surface heterogeneity, leading to significant pinning of the contact line. The Cu_SHPL surface exhibited superhydrophilic behavior, with water spreading completely across the surface as shown in Figure 6a. This is indicative of the Wenzel wetting state, where the increased surface roughness amplifies the intrinsic hydrophilicity of copper oxide, leading to complete wetting [24,25]. Upon PFOTES functionalization, the Cu_HPB surface showed an increased contact angle, indicating enhanced hydrophobicity; however, the presence of moderate contact angle hysteresis suggested that some pinning sites remained, resulting in only a partial transition toward ideal hydrophobic behavior. In contrast, the nanostructured and silane-coated Cu_SHPB surface exhibited both a very high contact angle and low hysteresis, as shown in Figure 7. This combination is characteristic of the Cassie–Baxter wetting regime, where the interplay between hierarchical surface roughness and low-surface-energy chemistry significantly reduces the solid–liquid contact area (see Figure 6), effectively suppressing pinning and allowing water droplets to roll off the surface [26].

Low contact angle hysteresis on these superhydrophobic surfaces is directly linked to reduced contact line pinning and improved droplet mobility, which are essential for reliable self-cleaning performance. The progression of wettability observed across the different samples highlights the critical role of combining nanoscale roughness with chemical functionalization to achieve and sustain the Cassie–Baxter state.

**Figure 8:**
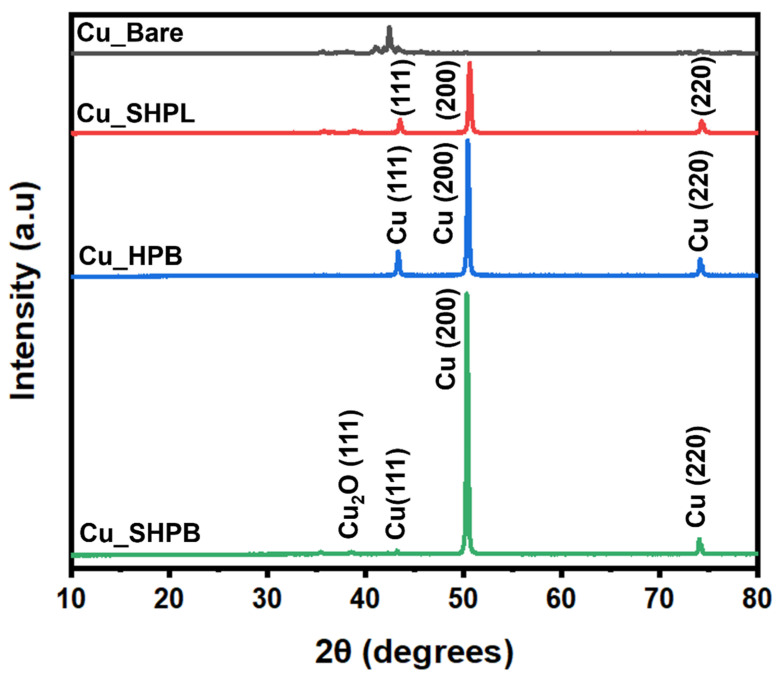
X-ray diffraction (XRD) patterns of various copper samples with phase assignments.

Figure 8 presents the XRD patterns for both uncoated and coated samples. The uncoated sample exhibits a peak at approximately 43.3°, which can be attributed to the (110) crystallographic plane of Cu (fcc), confirming the presence of the copper phase. However, all three coated samples display distinct Bragg reflections at 2θ values 43.4°, 50.4°, and 74.1°, which correspond to the crystallographic planes (111), (200), and (220) of the cubic structure of Cu (ICDD card no. 04-0836). For Cu_SHPB, less intense diffraction peaks are observed at 2θ values of 35.5° and 38.7°, which are associated with the planes (−111) and (111), respectively, revealing the presence of monoclinic CuO [22]. Each diffraction peak corresponding to the identified phases (Cu and CuO) is individually labeled in Figure 8 for clarity. The XRD analysis demonstrates the successful deposition of crystalline Cu with a strong (200) texture on the coated samples, while also revealing the formation of minor monoclinic CuO phase in the Cu_SHPL and Cu_SHPB. The clear distinction between the diffraction patterns of the uncoated and coated samples highlights the effectiveness of the coating process in modifying the surface phase composition and influencing the crystallographic orientation.

### 3.2. Electrochemical Corrosion Behavior

Electrochemical impedance spectroscopy (EIS) was performed in a 3.5 wt% NaCl solution to evaluate and compare the corrosion inhibition behavior of the engineered copper surfaces.

**Figure 9:**
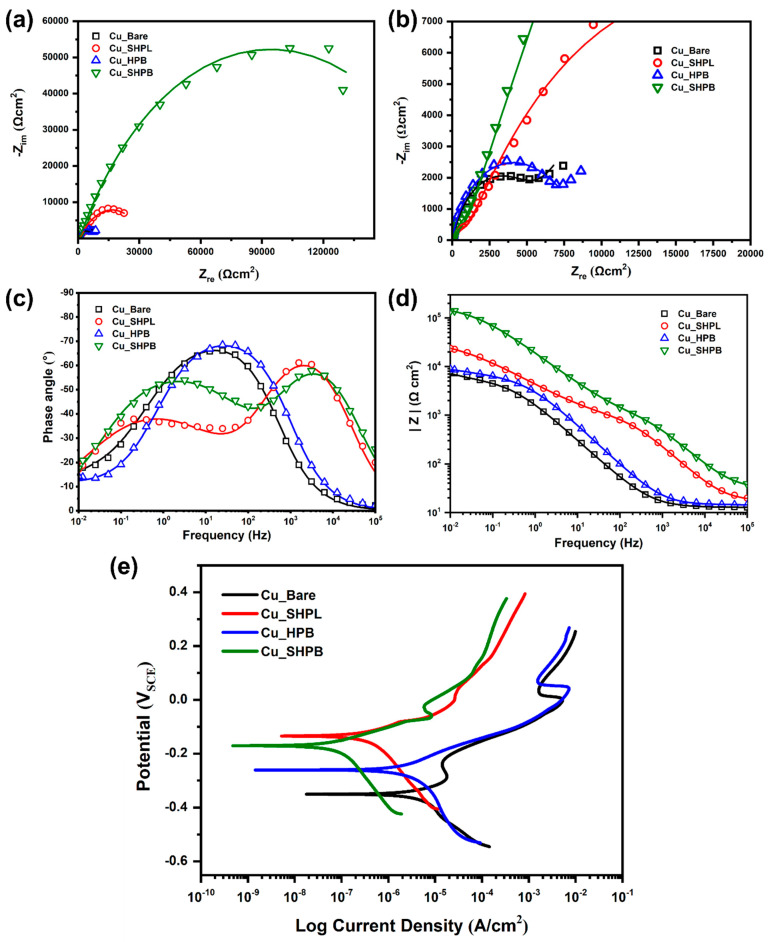
Electrochemical characterization of copper surfaces at different modification stages: (**a**) Nyquist plots from EIS showing impedance responses; (**b**) enlarged Nyquist plots highlighting low-impedance regions; (**c**) Bode phase angle plots illustrating phase response across frequencies; (**d**) Bode magnitude plots comparing impedance magnitude; and (**e**) potentiodynamic polarization curves demonstrating differences in corrosion potential and current density.

Nyquist plots (Figure 9a,b) reveal that the Cu_SHPB sample exhibits the largest semicircle diameter, corresponding to the highest polarization resistance (R_ct_), and thus the most effective barrier to charge transfer and corrosion. The Cu_SHPL sample demonstrates improved inhibition compared to Cu_Bare, but is less effective than Cu_SHPB. Among the coated samples, Cu_HPB shows the least inhibition effect. The Cu_SHPB sample achieved an inhibition efficiency of 97.1% as shown in Table 1, indicating excellent corrosion protection.

Bode plots (Figure 9c,d) further confirm these findings, with the Cu_SHPB sample showing the highest impedance at low frequencies, reflecting superior polarization resistance and barrier properties. Higher phase angle values, especially at intermediary and low frequencies, are indicative of enhanced capacitive behavior. This occurs when the surface modification effectively suppresses charge transfer processes and promotes the formation of a protective barrier layer. For the nanostructured and silane-coated copper (Cu_SHPB), phase angles approached across a broad frequency range, reflecting the presence of an intact, highly capacitive interface that impedes corrosion-related reactions. In contrast, bare copper exhibited much lower phase angles, consistent with a more pronounced resistive (faradaic) response due to active corrosion.

Intermediate phase angle behavior was observed for the nanostructured (Cu_SHPL) and silane-coated (Cu_HPB) samples, aligning with their moderate corrosion resistance compared to the Cu_SHPB surface.

Potentiodynamic polarization (PDP) curves (Figure 9e) were recorded for each sample to further assess corrosion behavior. The corrosion potential (E_corr_) and corrosion current density (icorr) were extracted using the Tafel extrapolation method, with the results summarized in Table 2. A positive shift in E_corr_ and a reduction in i_corr_ were observed for coated samples, indicating decreased corrosion activity. As expected, the corrosion rate trend closely follows that of i_corr_, with the Cu_SHPB sample exhibiting the lowest current density and corrosion rate, confirming its superior corrosion resistance [27].

### 3.3. Corrosion Test (Salt Spray Test)

**Figure 10:**
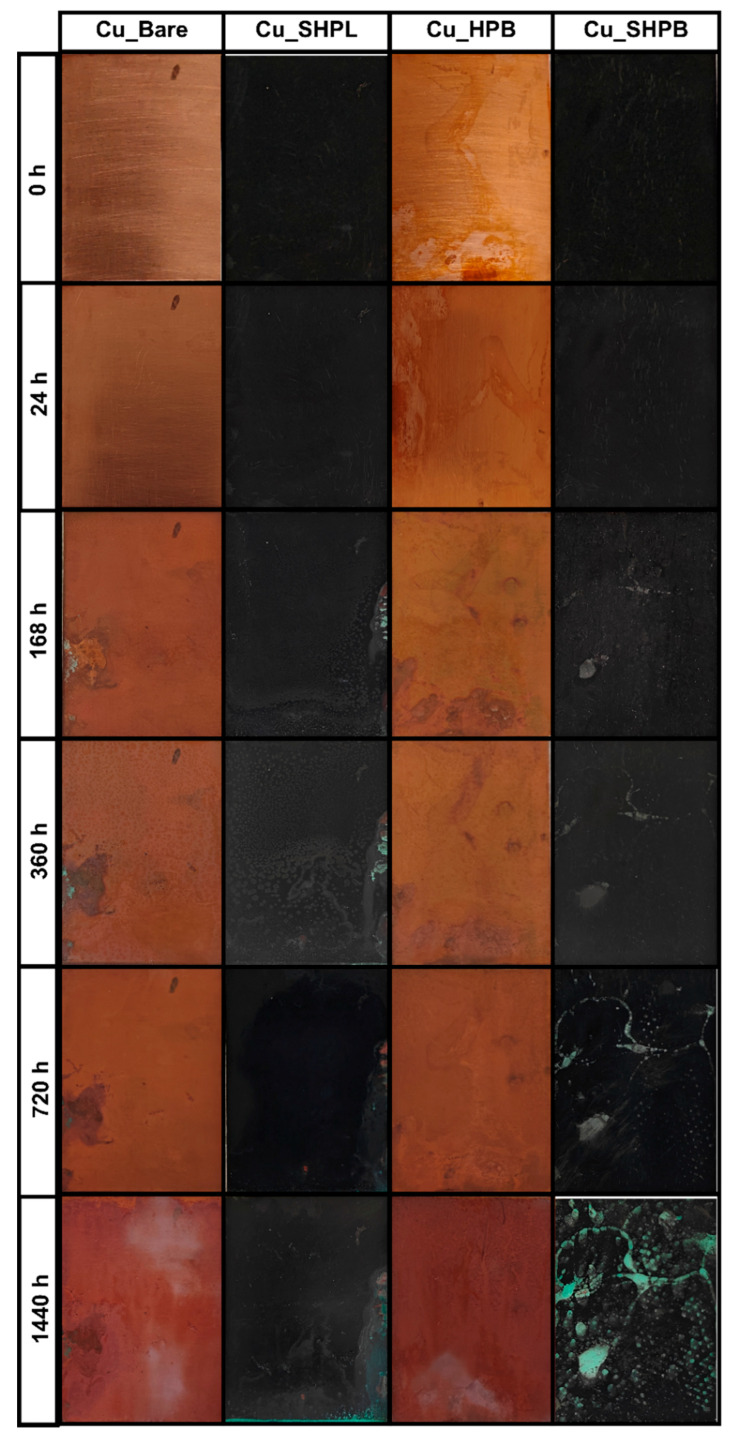
Images of the salt spray test for various copper surfaces after 1440 h of exposure to a 5 wt% NaCl environment.

**Figure 11:**
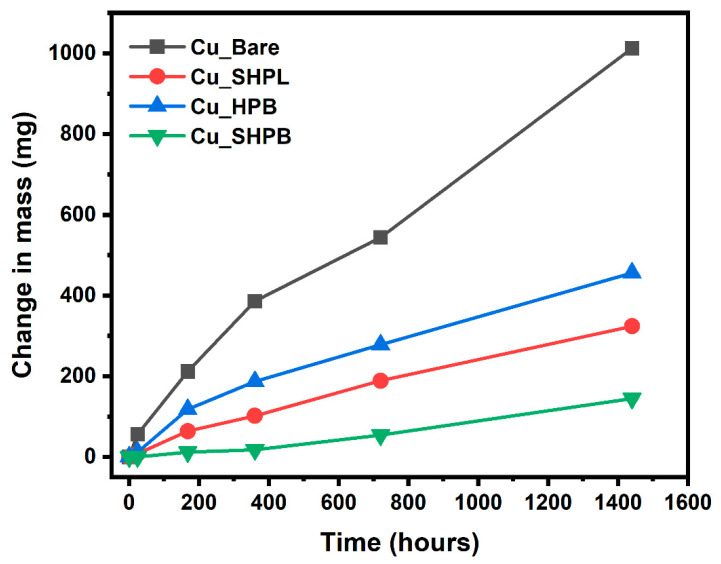
Change in mass of various samples as a function of salt spray exposure time.

Figure 10 provides a clear visual representation of the corrosion progression observed on copper surfaces with different surface treatments during prolonged salt spray exposure, highlighting the distinct protective mechanisms and failure points associated with each modification. The Cu_Bare sample developed green corrosion products within 168 h, which progressively transformed into a widespread reddish-brown oxide layer, ultimately covering the entire surface and indicating severe, unchecked corrosion. In contrast, the Cu_SHPL initially exhibited localized green and white stains, but these diminished over time as a stable oxide layer formed, resulting in a relatively unchanged, darkened appearance that suggests the development of a passivating barrier limiting further corrosion. The Cu_HPB showed reddish-brown stains after 168 h, with gradual but contained discoloration, reflecting that the hydrophobic coating slowed but did not fully prevent oxidation. Gravimetric salt spray testing revealed substantial differences in the change in mass among the variously modified copper surfaces, as shown in Figure 11. After 1440 h of exposure, the bare copper exhibited the highest corrosion rate at 2.51 mm/year, consistent with its susceptibility to chloride-induced degradation in saline environments. In contrast, the nanostructured surface (Cu_SHPL) and the silane-coated surface (Cu_HPB) showed significantly reduced corrosion rates of 0.80 mm/year and 1.13 mm/year, respectively. The most pronounced protection was observed for the combined nanostructured and silane-coated surface (Cu_SHPB), which demonstrated a corrosion rate of only 0.36 mm/year.

The most robust performance was demonstrated by the Cu_SHPB, which remained largely unchanged except for minor white stains in the early stages and only developed isolated green corrosion spots after extended exposure, with minimal spread even after 1440 h. This outcome underscores the synergistic effect of combining hierarchical surface roughness with chemical functionalization, as this integrated approach effectively delayed the onset and restricted the progression of corrosion, providing superior long-term protection for copper surfaces in aggressive saline environments [28].

## 4. Conclusions

This study demonstrates an effective and accessible strategy for engineering superhydrophobic, corrosion-resistant copper surfaces through the combination of wet chemical nanostructuring and silane-based chemical vapor deposition.

1. The resulting surfaces exhibit hierarchical CuO nanostructures conformally coated with a low-surface-energy silane, yielding water repellency with a contact angle of ~170° and a low contact angle hysteresis of ~3°, and robust resistance to salt-induced corrosion. Surface analyses confirm that the hierarchical morphology is preserved throughout the modification process.
2. Wettability measurements reveal that both roughness and chemical functionalization are essential for achieving the Cassie–Baxter wetting regime and droplet mobility.
3. Salt spray testing further validates the enhanced durability and protective performance of the fabricated surfaces.
4. The approach outlined here provides a scalable and reproducible platform for the development of advanced copper interfaces with applications in self-cleaning, anti-fouling, and long-lasting protective coatings.
5. This work contributes valuable insights into the design of next-generation functional materials [29,30].

## Figures and Tables

**Table 1:** Electrochemical impedance spectroscopy (EIS) and polarization parameters for all samples.

Sample	R_s_	R_f_	R_ct_	CPE_f_	CPE_dl_	m	n	R_p_ (Ω cm^2^)	IE (%)
	(Ω cm^2^)			(F/cm^2^)					
UC	12.77	-	5421	-	1.13 × 10^−4^	-	0.76	5421	-
SHPL	16.7	1125	31,240	3.87 × 10^−6^	8.54 × 10^−5^	0.80	0.59	32,365	82.6
HPB	13.57	755	8776	1.32 × 10^−4^	1.46 × 10^−4^	0.89	0.45	9531	38.2
SHPB	32.1	1226	187,204	1.46 × 10^−6^	1.58 × 10^−5^	0.81	0.64	188,430	97.1

**Table 2:** Potentiodynamic Polarization (PDP) Parameters and Calculated Corrosion Rates for all samples.

Sample	E_corr_ (V)	i_corr_ (µA/cm^2^)	Corrosion Rate (MPY)	IE (%)
UC	−0.350	5.900	2.720	-
SHPL	−0.134	0.560	0.258	90.5
HPB	−0.261	1.290	0.595	78.1
SHPB	−0.171	0.082	0.038	98.6

## Data Availability

The original contributions presented in this study are included in the article. Further inquiries can be directed to the corresponding authors.
